# An Analysis of Genetic Diversity and Inbreeding in *Wuchereria bancrofti*: Implications for the Spread and Detection of Drug Resistance

**DOI:** 10.1371/journal.pntd.0000211

**Published:** 2008-04-02

**Authors:** Thomas S. Churcher, Anne E. Schwab, Roger K. Prichard, María-Gloria Basáñez

**Affiliations:** 1 Department of Infectious Disease Epidemiology, St. Mary's Campus, Imperial College London, London, United Kingdom; 2 Institute of Parasitology, McGill University, Sainte-Anne-de-Bellevue, Quebec, Canada; Australian Centre for International and Tropical Health, Australia

## Abstract

Estimates of genetic diversity in helminth infections of humans often have to rely on genotyping (immature) parasite transmission stages instead of adult worms. Here we analyse the results of one such study investigating a single polymorphic locus (a change at position 200 of the β-tubulin gene) in microfilariae of the lymphatic filarial parasite *Wuchereria bancrofti*. The presence of this genetic change has been implicated in benzimidazole resistance in parasitic nematodes of farmed ruminants. Microfilariae were obtained from patients of three West African villages, two of which were sampled prior to the introduction of mass drug administration. An individual-based stochastic model was developed showing that a wide range of allele frequencies in the adult worm populations could have generated the observed microfilarial genetic diversity. This suggests that appropriate theoretical null models are required in order to interpret studies that genotype transmission stages. Wright's hierarchical *F*-statistic was used to investigate the population structure in *W. bancrofti* microfilariae and showed significant deficiency of heterozygotes compared to the Hardy-Weinberg equilibrium; this may be partially caused by a high degree of parasite genetic differentiation between hosts. Studies seeking to quantify accurately the genetic diversity of helminth populations by analysing transmission stages should increase their sample size to account for the variability in allele frequency between different parasite life-stages. Helminth genetic differentiation between hosts and non-random mating will also increase the number of hosts (and the number of samples per host) that need to be genotyped, and could enhance the rate of spread of anthelmintic resistance.

## Author Summary

During the last decade, there has been a substantial increase in the use of mass drug administration to reduce the disease caused by parasitic worms. With so many people regularly receiving treatment, there is a risk that drug resistance may develop. As a result, the number of studies looking for genetic markers of drug resistance has increased noticeably. In this paper we analyse the results of one such study that investigated the presence of genes associated with drug resistance in parasites responsible for elephantiasis. This study, like many other studies of human parasitic infections, relies on analysing parasite immature stages (such as eggs) because the adult worms are often inaccessible within the human body. Using computer models we show how the gene frequency in the immature stages may vary from that in the adult worm population. Parasites with these markers for drug resistance might also be unevenly distributed across the host population even prior to treatment. This may increase the spread of drug resistance and make it harder to detect. We suggest that studies conducted only on parasite immature stages should be interpreted with caution and should carefully consider the number of people and the number of parasites they analyse.

## Introduction

In recent years there has been a substantial increase in the use of mass drug administration (MDA) to reduce the morbidity associated with helminth infections of humans [1], increasing the probability that anthelmintic resistance may become a public health concern in the future. One such annual MDA programme is the Global Programme to Eliminate Lymphatic Filariasis (GPELF) which, in 2005, treated over 145 million people with albendazole (a broad spectrum benzimidazole anthelmintic) in combination with either ivermectin or diethylcarbamazine [2]. GPELF targets mainly *Wuchereria bancrofti*, the most widely distributed of the filarial parasites of humans.

Sensitive molecular assays are required to detect the presence of anthelmintic resistance before widespread treatment failure is apparent, drug resistance becomes disseminated and disease control is jeopardised [3]. Surveys of helminth parasites of humans are being conducted to establish whether genetic changes at certain polymorphic loci (associated with resistance to the same or related drugs used against veterinary helminths), are present in these populations and subject to detectable selection under chemotherapeutic pressure [4]–[13]. A phenylalanine to tyrosine substitution at position 200 on the β-tubulin isotype 1 molecule has been identified in a number of helminth parasites of farmed ruminants including *Haemonchus contortus* [14],[15], *Cooperia oncophora* [16], and *Teladorsagia circumcincta* [17] and is associated with benzimidazole (BZ) resistance in these species. Worryingly, this genetic change has also been identified in *W. bancrofti* [13], though the phenotypic studies relating the substitution to a decreased albendazole efficacy have not been undertaken in this species. To aid clarity the two alleles at position 200 on the β-tubulin isotype 1 molecule shall be referred to as allele *F* (phenylalanine) for susceptibility and allele *Y* (tyrosine) for putative resistance.

Inbreeding, the mating of related individuals, influences parasite genotype distribution and can affect the selection of adaptive traits. Facets of a species' biology may cause parasite inbreeding, such as population structure or assortative mating (when mate choice is determined by phenotype). Parasite allele frequency can differ between infrapopulations (the populations of parasites within individual hosts) due to the ecology of the infection or through the random nature of infection events (all groups may have an equal probability of having a rare allele, but actual numbers may vary between groups by chance). Helminth parasites have a particularly subdivided population structure as adult worms are confined within their definitive host, and only able to mate with other worms that belong to the same infrapopulation. The population genetic structure of most helminth species remains unknown. The few studies that have been undertaken indicate that whilst some species appear to have no apparent genetic structure others exhibit a high degree of parasite genetic differentiation between hosts [18]. The degree of genetic differentiation in the parasite infrapopulation can shed insight into the microepidemiology of parasite transmission [19]–[23]. Infrapopulation genetic differentiation will also influence helminth population genetics as it causes a reduction in the frequency of heterozygote offspring, a phenomenon known as the Wahlund effect [24].

Studies investigating the inheritance of benzimidazole resistance are lacking, though evidence indicates that thiabendazole resistance in *H. contortus* may be a semi-dominant trait [25]. Other authors have postulated that alleles conferring anthelmintic resistance, including allele *Y*, are likely to be recessive [17],[26], which would make heterozygote worms susceptible to treatment. If an allele conferring drug resistance is recessive, excess parasite homozygosity will increase the probability that a resistance allele will survive treatment. This has been shown using genetic metapopulation models investigating nematodes of grazing animals; these models indicate that the spread of rare recessive genes is promoted by hosts accumulating multiple related infections simultaneously [27],[28]. The degree of parasite genetic differentiation among hosts can be quantified using *F_ST_* (or related analogues; see [18] and references therein).

The adult stages of the majority of parasitic helminths of humans cannot be obtained routinely for direct investigation, so genetic surveys (including those investigating drug resistance) resort to sampling transmission stages, i.e. those (immature) life-stages that gain access to the environment to be transmitted to and from hosts or through vectors [13], [29]–[32]. However, the results of these surveys should be interpreted with caution, as the underlying allele frequency of the adult worm population may differ from the allele frequency of the sampled transmission stages. Variations in transmission stage allele frequency and genotype distribution could be generated randomly or be a product of the parasite's spatial structure and life-history traits. For example, population subdivision will cause random variation in adult worm allele frequencies between hosts at low parasite densities. Filarial parasites have separate sexes and are thought to be polygamous [33], which may accentuate the variability in microfilarial allele frequency, e.g. a rare allele may be highly over-represented in the subsequent generation if, by chance, a male worm with this allele inhabits a host harbouring females but no other males. In addition, the inherent random sampling of gametes during sexual reproduction [34], and the overdispersed distribution of parasite numbers among hosts [35] may cause the allele frequency and genotype distribution to vary by chance from generation to generation.

This paper analyses population genetic data collected for a study by Schwab *et al.* [13] who identified the presence of the β-tubulin allele *Y* in populations of *W. bancrofti*. Firstly, the extent of parasite inbreeding is estimated from *W. bancrofti* microfilarial samples taken from patients in Burkina Faso, West Africa. Samples were obtained from different villages, some of which had received a single round of MDA with ivermectin and albendazole, under the auspices of the GPELF. Secondly, an individual-based stochastic model is presented which simulates microfilarial genetic diversity from adult worm allele frequencies. The model generates sample allele and genotype frequencies using the same number of hosts, and the same number of microfilariae per host as in Schwab *et al.* [13]. This model is then used to assess whether the observed level of parasite inbreeding is the result of a sampling artefact or a true biological phenomenon. Finally, the model is used to assess the likely range of adult worm allele frequencies which could have given rise to the observed microfilarial data, providing some insight into how genetic surveys which sample transmission stages should be interpreted. We discuss the implications of our results in terms of the development and detection of anthelmintic resistance.

## Materials and Methods

### Sampled data

Table 1 summarises the data collected for the study by Schwab *et al.* [13] and indicates the number of microfilariae and hosts sampled. The village of Gora was removed from the *F*-statistic analysis since only one host was sampled in this village. In some hosts it was possible to genotype only a few microfilariae, increasing the uncertainty associated with estimation of underlying infrapopulation allele frequencies in these hosts. Results are grouped according to parasite treatment history. The average frequencies of allele *Y* in microfilarial samples from untreated and treated hosts were 0.26 and 0.60, respectively [13]. The degree of parasite heterozygosity (the proportion of microfilariae with the heterozygote genotype) is estimated for each village. The table also indicates the deviation of each population from the Hardy-Weinberg Equilibrium (HWE), which gives the proportion of heterozygote microfilariae that would be expected in a randomly mating population. This reveals a strong deficit of heterozygotes in all three populations.

**Table 1:** Summary of the genetic survey conducted on *Wuchereria bancrofti* microfilariae from Burkina Faso of genetic changes at the β-tubulin locus associated with benzimidazole resistance (in nematodes of ruminants).

Village	No. hosts sampled	Mean no. of microfilariae genotyped per host (range)	Mean microfilaraemia per 20 µl blood (range)	Sample (microfilarial) resistance allele frequency, 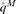	Sample and [expected] heterozygosity
Untreated villages					
TANGONKO	16	9.6 (1, 15)	323 (162, 703)	0.28	0.20 [0.40]
BADONGO	14	6.6 (1, 10)	212 (60, 845)	0.23	0.24 [0.35]
Village that had received one round of chemotherapy (albendazole+ivermectin)					
PERIGBAN	13	8.5 (3, 12)	35 (18, 86)	0.62	0.27 [0.47]

Results were presented by Schwab *et al.* [13]. The range of microfilarial samples obtained per host is given in brackets. The expected microfilariae heterozygosity according to the Hardy-Weinberg equilibrium is given in square brackets.

In this paper, we refer to two different types of allele frequency: (1) the underlying frequency of the allele putatively associated with BZ resistance, with *q^l^* denoting the allele frequency of the entire parasite population of a given locality, and (2) the parasite allele frequency within the host population that is sampled, denoted by *^H^q^l^*. The superscript *l* denotes the parasite life-stage under investigation, be it microfilariae (*l = M*) or adult worms (*l = W*), and *H* denotes definitive host. The allele frequency estimated from the sample, 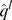, may not correspond to the true underlying allele frequency, *q^l^*, either because the hosts sampled are not representative of the whole host population, or because the parasites genotyped do not represent adequately the allele frequency within the host.

### Estimating parasite inbreeding

By genotyping transmission stages before they leave the definitive host prior to the introduction of mass chemotherapy, insight can be gained into the different causes of microfilarial excess homozygosity. If it is assumed that the number of microfilariae produced, their survival, and their probability of being sampled are independent of their genotype (as we do in the null model), it can be assumed that deviation from the HWE may be the result of non-random mating. If the locus being investigated is not under selection, the excess microfilarial homozygosity will most likely be the result of either infrapopulation genetic differentiation or non-random parasite mating within hosts. Genotyping transmission stages would allow the relative contributions of each of these two sources of inbreeding to be estimated. The variation in the allele frequency between hosts will account for some of the excess homozygosity whilst deviation from the HWE in the microfilariae within an individual host will indicate possible non-random mating within the infrapopulation.

The Wright's hierarchical *F*-statistic is used to investigate the correlation of parasite genes within and between human hosts [29]–[31],[36]. It is assumed that the infrapopulation is the first hierarchical group in the parasite population, and *F_IS_* is defined as the correlation of genes between microfilariae within the infrapopulation; 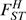, as the correlation of microfilarial genes between different hosts living in the same village; 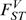, as the correlation of microfilarial genes between different villages within the overall microfilarial population; and *F_IT_*, as the correlation of genes between individual microfilariae relative to the overall microfilarial population of the region. The different inbreeding terms introduced are summarized in Table 2. A value of *F_IS_* is significantly greater than zero points towards adult worm non-random mating, 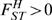 indicates variation in worm allele frequency between hosts, and 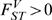 suggests differences in the worm allele frequency between villages. The same statistical frameworks used to estimate Wright's *F*-statistic were employed here, taking into account variable sample sizes [34]. Estimates of the 95% confidence intervals for *F_IS_*, 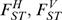 and *F_IT_*, were generated by bootstrapping simultaneously worms within each host and bootstrapping over hosts within each village [37]. *F-*statistics, and their associated uncertainty, were calculated for each village.

### Modelling the allele frequency and genotype distribution of microfilariae

A dioecious adult worm helminth population with a 1:1 male to female ratio was randomly generated for a given mean number of worms per host and degree of parasite overdispersion (as determined by the *k* parameter of the negative binomial distribution, parameterized following [35]). Each adult worm infrapopulation was randomly allocated an allele frequency, as analysis of pre-treatment data did not detect any significant relationship between the host's frequency of allele *Y* and microfilarial burden. The adult worm allele frequency of each host was randomly selected according to the given underlying allele frequency, *q^W^*, and the degree of parasite genetic differentiation between hosts, 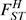. For a description of a method for generating the distribution of allele frequencies in a subdivided population using the beta distribution [38], see Porter [39].

It is again assumed that microfilarial production and survival is independent of genotype, allowing a microfilarial population for each host *i* to be generated according to the size and allele frequency of the adult worm infrapopulation. Worms were assumed to be polygamous; implying that if only one male parasite were present within a host, all fertile females within that infrapopulation would be mated. The number of microfilariae produced by each parasite infrapopulation was assumed to be proportional to the number of fertilised females within that host. It was also assumed that gametes separate independently and re-assort according to the degree of non-random mating (*F_IS_*). The probability with which a microfilaria within host *i*, will be of genotype *j* is denoted 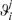, and given by the equations,
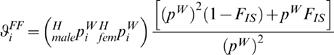
(1)

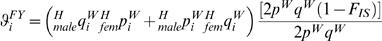
(2)

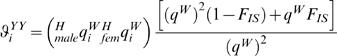
(3)where 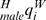 and 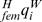 are, respectively, the frequency of allele *Y* in the male and female adult worms within host *i*, and 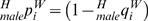 and 
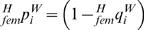
 are the corresponding susceptible allele *F* frequencies. To allow random stochastic fluctuations in genotype distribution, the actual number of microfilariae in host *i* with genotype *j* follows a binomial distribution, with the number of trials being equal to the number of microfilariae produced by host *i*, with genotype probability equal to 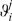.

Microfilarial allele frequencies and genotype distributions were generated by sampling a specific number of microfilariae from the generated hypothetical population according to the sampling scheme used in Schwab *et al.* [13]. The exact number of samples taken from each of the 30 hosts was: 11, 10, 15, 9, 11, 9, 13, 10, 10, 7, 10, 10, 7, 1, 11, 9, 1, 7, 4, 1, 10, 9, 8, 6, 4, 6, 9, 10, 10, 8, for a total of 246 microfilariae. Analysis of pre-treatment data had indicated that the number of samples taken from each host by Schwab *et al.* [13] was independent of host microfilaraemia and host allele frequency, allowing the number of microfilariae sampled per host to be randomly allocated. The program code for the simulations implemented was written in C++ and run 100,000 times, with each run generating a new helminth population and genotype distribution from which 95% confidence limits (95% CL) were calculated.

The model was parameterised for the untreated villages of Tangonko and Badongo, Burkina Faso, which had an initial prevalence of microfilaraemia of 25%. The mean adult worm burden was estimated from observed microfilarial counts using the functional relationship given in the deterministic model EPIFIL (see original formulation and parameter values in Norman *et al*. [40]), giving a mean adult worm burden of 13.5 host^−1^. The degree of adult worm overdispersion was estimated from the recorded microfilarial prevalence (taken here as a proxy for the prevalence of adult worms producing microfilariae) and the mean adult worm burden, using the prevalence vs. intensity relationship that derives from assuming a negative binomial distribution of worms among hosts [35], yielding a *k* value of 0.07. The model outlined above will only be valid for comparisons against the pre-treatment data, since chemotherapy is known to impede microfilarial production and / or survival [41].

The null model assumes that mating is random between male and female worms within each infrapopulation and that allele *Y* is randomly distributed across hosts, i.e. 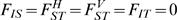. Results of the inbreeding analysis can be incorporated into the individual-based model described in equations (1) to (3) to explore the range of adult worm allele frequencies which can give rise to the observed microfilarial data.

## Results

The observed microfilarial genotype distribution was found to deviate from HWE. Villages with no history of mass anthelmintic chemotherapy had an overall inbreeding coefficient of *F_IT_* = 0.44 (95% CL = 0.17, 0.68), indicating strong inbreeding. Fifteen percent of the microfilariae were found to be homozygous for allele *Y*, an estimate 2.3 times higher than would be expected in a random mating parasite population. Results indicate the occurrence of a significant degree of genetic differentiation in worm allele frequency among the host population 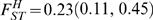. Infrapopulation allele *Y* frequency, 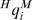, varied from 0 to 0.77 in the villages with no history of treatment, indicating an increase in microfilarial homozygosity of 60% above HWE. The results also suggest a degree of non-random mating within hosts measured by *F_IS_* = 0.29 (−0.09, 0.54), which is however is not significantly greater than zero. No difference was observed in the microfilarial allele frequency between the two treatment-naïve villages 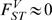.

**Figure 1:**
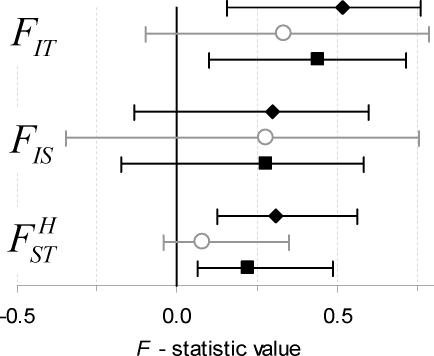
Estimates of Wright's *F*-statistics in *Wuchereria bancrofti* for the pre-treatment villages of Tangonko (black diamonds), Badongo (grey open circles) and for the treated village of Perigban (black squares), which received one round of chemotherapy (albendazole+ivermectin). The error bars are the 95% confidence intervals. *F_IT_* estimates the total degree of parasite inbreeding; *F_IS_* describes the level of non-random mating within the infrapopulation; and 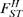 shows the variation in microfilarial allele frequency within the host subpopulation (village).

The data from the two treatment-naïve villages of Tangonko and Badongo were analysed separately. Both showed a high level of microfilarial homozygosity, with overall inbreeding coefficient of *F_IT_* = 0.51 (0.16, 0.76) and *F_IT_* = 0.33 (−0.10, 0.78), respectively (Figure 1). The degree of parasite genetic differentiation between hosts varied between the two villages, though the difference was not statistically significant (*p* = 0.38, calculated from the square of the normalized difference in *F_ST_* estimates [42]). For the purpose of the following analysis the two treatment-naïve villages have been grouped together to increase the study sample size. A similar degree of parasite inbreeding was observed in the village of Perigban which had received one round of MDA.

**Figure 2:**
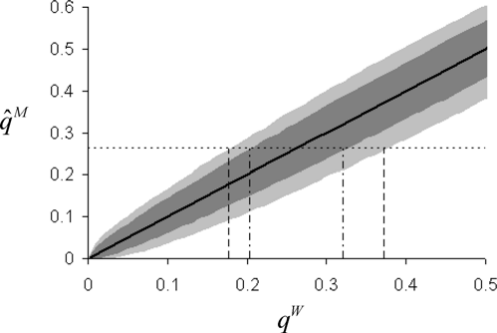
The impact of inbreeding on the relationship between the sample microfilarial allele frequencies, 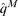, and the (inferred) underlying adult worm allele frequency, *q^W^*, for the substitution at codon 200 of the β-tubulin gene in *W. bancrofti*. The figure shows 95% confidence intervals for a population with no excess inbreeding (the null model, dark grey shaded area), and a population with the observed levels of inbreeding (*F_IS_* = 0.28, 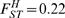, light grey shaded area). Simulations are based on the same sampling scheme used in Burkina Faso [13]. The thick black solid line indicates the mean result for both models. The observed pre-treatment microfilarial allele frequency (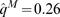; black thin, horizontal dotted line) was compared to simulation results to indicate the possible range of adult worm allele frequencies which could have given rise to the West African data. The null model (black vertical dotted-dashed lines) indicated values of *q^W^* ranging from 0.21 to 0.32 compared to the inbred model (*F_IS_* = 0.28, 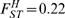, black vertical dashed lines), which gave values of *q^W^* between 0.18 and 0.37.

Parasite inbreeding increases the range of underlying adult worm allele *Y* frequencies, *q^W^*, which can give rise to the observed microfilarial allele *Y* frequency of 0.26 (Figure 2). Results from the null model, where mating was assumed to be random and allele *Y* is randomly distributed amongst hosts, indicate that *q^W^* in the untreated villages of Tangonko and Badongo could range from 0.21 to 0.32. If we use the excess inbreeding estimate reported in pre-treatment villages (*F_IT_* = 0.44), then model simulations suggest that *q^W^* could range from 0.18 to 0.37.

**Figure 3:**
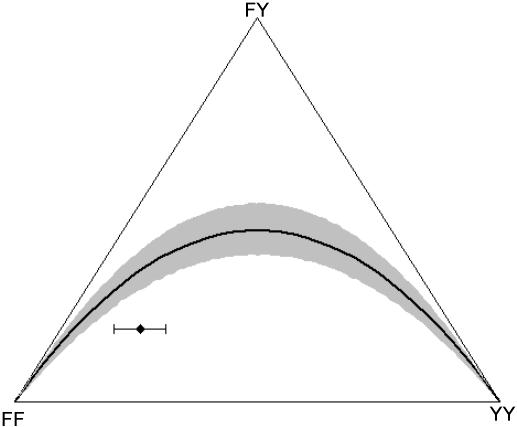
De Finetti diagram showing the genotype distribution of *W. bancrofti* microfilariae generated from a given underlying adult worm allele frequency, *q^W^*, taken from villages prior to the introduction of chemotherapy. A full explanation of the De Finetti diagram is given in [24]. The black diamond represents the value originating from the observed data (with 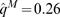, and *F_IT_* = 0.44), and the error bars indicate the uncertainty in genotype distribution stemming from the values of *q^W^* (0.21, 0.32) that were estimated from the null (random) model (Figure 2). Y indicates the allele coding for tyrosine at position 200 of β-tubulin that is associated with benzimidazole (BZ) resistance in nematodes of livestock, and F denotes the allele (coding for phenylalanine) indicative of BZ susceptibility. The solid-black curve represents the Hardy-Weinberg equilibrium (HWE). The null model generating microfilarial allele frequencies (see text) was used to investigate the range of sample microfilarial genotype distributions that could be obtained from a population exhibiting no excess inbreeding (i.e. assuming that the underlying adult parasite population would have values of 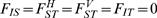). Simulations mimic the same sampling scheme described in Schwab *et al*. The observed microfilarial genotype distribution falls outside the 95% confidence interval range (grey shaded area surrounding the HWE curve) generated by the null model, despite the uncertainty in the underlying *q^W^* estimates, indicating strong parasite inbreeding even before introduction of antifilarial combination therapy.

The microfilarial genotype diversity model indicates that the observed homozygosity is unlikely to be solely a result of genetic sampling, demographic stochasticity, population subdivision, or the sampling scheme employed, suggesting that true biological mechanisms are operating in the parasite population even before the introduction of anthelmintic therapy. Figure 2 indicates the range of likely microfilarial genotype distributions that can be generated from a given *q^W^* value using the null (random) model. The observed excess homozygosity in the untreated villages was greater than the 95% confidence interval estimates generated by the null model (Figure 3). It is interesting to note the wide range of microfilarial genotype distributions that can be generated by the null model.

**Figure 4:**
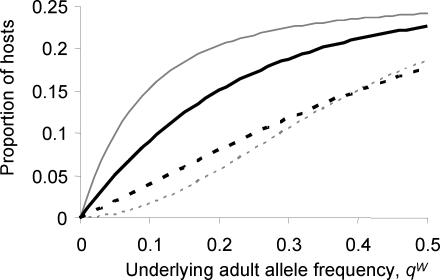
The impact of inbreeding on the relationship between the mean proportion of hosts harbouring microfilariae with one or two copies of allele *Y* and the (assumed) underlying adult worm allele frequency, *q^W^.* The figure compares the proportion of hosts exhibiting microfilariae with allele *Y* (i.e. both heterozygous and homozygous *YY* microfilariae, solid lines) with that of hosts which have only microfilariae with the homozygous *YY* genotype (broken lines). Model outcomes are compared for two hypothetical parasite populations; the former (thin grey lines) without excess inbreeding (generated by the null model), and the latter (thick black lines) with the levels of inbreeding (*F_IS_* = 0.28, 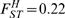) observed in the Burkina Faso data. Simulations used the same sampling scheme described in Schwab *et al.* [13] and assume an overall microfilarial prevalence of ∼25% (see text).

**Figure 5:**
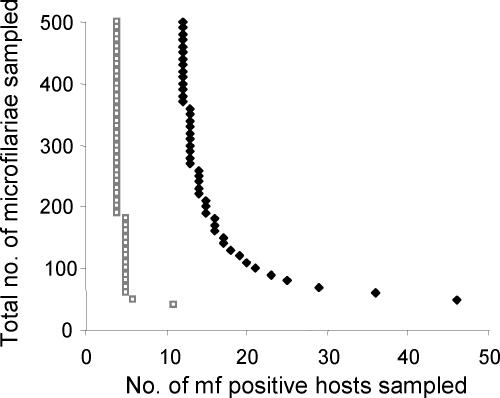
The impact of helminth inbreeding on the minimum number of microfilaria-positive hosts who should be sampled and the minimum number of microfilariae that should be genotyped to be 95% confident of detecting at least one rare allele. A randomly mating population (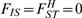, grey open squares) is compared to an inbred population (*F_IS_* = 0.28 and 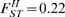, black diamonds). The underlying adult worm allele frequency of both populations is set at *q^W^* = 0.05. Each data point represents 100,000 runs of the stochastic model generating microfilarial allele frequencies. The number of microfilariae analysed per host is proportional to host microfilaraemia.

Despite the large increase in microfilarial homozygosity attributable to parasite inbreeding, there is only a modest increase in the prevalence of hosts who have microfilariae that are homozygous for allele *Y* (and therefore putatively resistant if the allele confers drug resistance were recessive, Figure 4). Parasite overdispersion reduces the number of hosts who are microfilaria-positive and concentrates allele *Y* into a small proportion of the host population. A high degree of parasite non-random mating and infrapopulation genetic differentiation increases the number of hosts (and the number of samples per host) that need to be sampled, in order to detect or quantify reliably parasite genetic diversity (Figure 4). The model is used to investigate how parasite inbreeding may influence the sampling scheme of genetic surveys seeking to identify the presence of a known marker for drug resistance (Figure 5). Results indicate that the observed level of parasite inbreeding markedly increases the minimum number of hosts, and the overall number of samples necessary to be 95% confident of detecting a rare allele. The sampling scheme used within Figure 5 assumes that the number of parasites genotyped per host is weighted by the host's microfilarial load. This improves the accuracy of allele frequency estimates by allowing heavily infected hosts to have a greater contribution to the sampled microfilarial population, something which is particularly important in overdispersed parasite populations.

**Figure 6:**
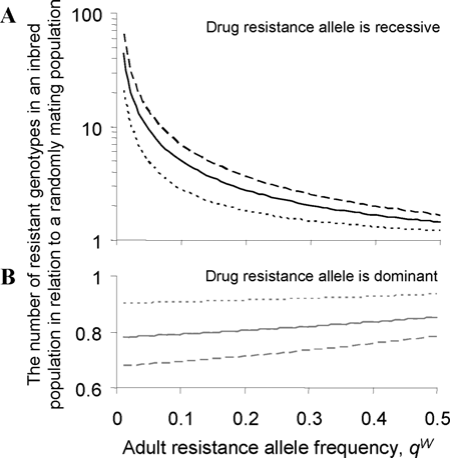
The impact of the observed level of parasite inbreeding on the production of resistant microfilariae. The graph gives the relative change in the number of resistant genotypes in an inbred parasite population compared to that in a population at HWE. Results are shown for different resistance allele frequencies. The graph assumes that a known resistance allele is either recessive (A), black lines, or dominant (B), grey lines. The inbreeding coefficients are those reported in Figure 1: mean result (*F_IT_* = 0.44, solid line); upper 95% confidence limit (*F_IT_* = 0.68, dashed line); lower 95% confidence limit (*F_IT_* = 0.17, dotted line). The relative change in the number of resistant genotypes caused by parasite inbreeding is estimated as 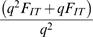 in (A) and 
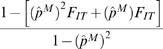
 in (B).

To date there is no phenotypic evidence that allele *Y* causes albendazole resistance in *W. bancrofti*. However, if an allele conferring drug resistance existed in populations of this parasite then the consequences on the spread of such an allele of parasite non-random mating and genetic differentiation between hosts will depend on the frequency and the relative dominance of the resistance allele. If the resistance allele were recessive, helminth inbreeding would greatly increase the probability that a parasite survives anthelmintic treatment. This is evident from Figure 6 which shows the influence of parasite inbreeding on the relative proportion of resistant genotypes for a given allele frequency. With a recessive resistance allele at a frequency of 0.05, the degree of inbreeding within the *W. bancrofti* population reported here, would on average increase the number of worms with the homozygote resistance genotype nine-fold. Conversely, if the resistance allele was dominant, inbreeding would reduce the probability that a parasite survives chemotherapy, as fewer worms would have the resistant allele (the deficiency of heterozygous parasites caused by parasite inbreeding will be greater than the increase in resistant homozygous worms).

**Table 2:** The extension of Wright's *F*-statistic to represent the hierarchical population structure of obligate parasites of humans, exemplified in this paper with *Wuchereria bancrofti* (adapted from [24] and [34]).

Symbol	Definition
*F_IT_*	Correlation of alleles within individual worms relative to alleles drawn at random from the overall worm population (total deviation from the Hardy-Weinberg Equilibrium)
*F_IS_*	Correlation of alleles within individual worms relative to alleles drawn at random from the parasite infrapopulation (within host non-random mating)
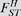	Correlation of parasite alleles within parasite infrapopulations relative to alleles drawn at random from parasites within the same village (parasite genetic differentiation between hosts within villages)
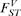	Correlation of parasite alleles within a village relative to alleles drawn at random from the overall worm population (parasite genetic differentiation between villages) 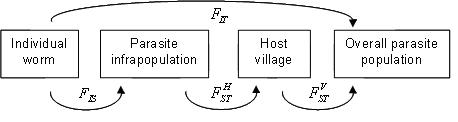

The table includes a graphical representation of the different *F*-statistics.

## Discussion

The genotype distribution of *W. bancrofti* microfilariae varied dramatically from the HWE prior to the introduction of MDA. The degree of excess homozygosity reported falls outside the range of values generated by the null model described in this paper, indicating a significant degree of parasite non-random mating. This may be caused, in part, by parasite genetic differentiation between hosts. The null model generates a wide range of microfilarial allele frequencies and genotype distributions indicating that caution should be exercised when interpreting results obtained by sampling solely transmission stages. Significant changes in the genetic diversity of microfilarial populations over time may not reflect a significant change in the underlying adult worm population. This result highlights the crucial importance of developing sound theoretical null models that enable helminth population genetics data to be interpreted adequately [43]. These models should take into account the uncertainty in outcomes, given the sampling scheme employed and the life-history traits of the parasite. A combination of sampling transmission stages and parasite inbreeding could cause estimates of the underlying adult worm allele frequency to be highly variable, increasing the number of samples that need to be genotyped in order to detect significant changes in the adult worm genome with time after introduction of chemotherapeutic pressure.

Producing a null model to assess the range of adult worm allele frequencies that could give rise to the microfilarial genetic diversity observed in villages having received treatment is complex and beyond the scope of this paper. A dynamic, full transmission model would be required that takes into account the pharmacodynamic properties of the drugs in combination and separately, as the effects of chemotherapy will influence microfilarial genetic diversity for a number of years after chemotherapy. As a result it is not possible to conclude whether adult worm genetic diversity differs between the villages that have and have not received MDA, even though their microfilarial populations differ significantly in their genetic diversity.

The results presented within this paper regarding the metapopulation dynamics of bancroftian filariasis stem from the analysis of a single nucleotide polymorphism in one gene. Further surveys, using multiple neutral polymorphic loci, are required to distinguish demographic and sampling effects from selective pressures [34]. If the allele of interest has been under selection then the observed genotype distribution could have been generated without the need for non-random parasite mating. The accuracy of the model developed here to derive microfilarial genetic diversity is limited by uncertainties regarding the biology of *W. bancrofti*. Results are dependent on our current ability to mimic adult worm burden and its distribution among hosts. Limitations inherent in the EPIFIL model, the presence of amicrofilaraemic yet circulating filarial antigen-positive infections, and possible heterogeneity in host immune responses could make adult worm burden estimates highly uncertain from microfilarial prevalence and intensity data. The relationship between the number of adult filariae and the rate of microfilarial production is likely to be complex and may depend on the immune responses elicited during the infection. The null model assumes a mean parasite intensity of 13.5 adult worms per host, though sensitivity analysis indicated that model results were relatively insensitive to small changes in parasite intensity around this value (sensitivity analysis ranged from 8.5 to 18.5 adult worms host^−1^, results not shown). Our conclusions are based on the adequacy of the null model, which may be improved by the inclusion of further biological detail. For example, recent evidence suggests a possible association between β-tubulin genotype in the related filarial parasite, *Onchocerca volvulus*, and female worm fertility [9],[10], suggesting a cost of resistance. Whilst the same gene has been analyzed in the current study, it is not known whether a similar relationship between genotype and fertility applies to *W. bancrofti*. If this were the case then the conclusions drawn regarding the causes of the observed genotype distribution should be treated with caution. Although no differences were seen in genotype frequency between the two pre-treatment villages studied, additional baseline surveys (prior to the start of MDA) would be required before firm conclusions regarding the true underlying frequency of allele *Y* in pre-treatment *W. bancrofti* populations can be drawn.

Notwithstanding the fact that the *F*-statistic provides a phenomenological tool rather than a mechanistic measure of inbreeding (and therefore does not describe the biological processes generating excess homozygosity), we proceed to propose some likely causes for the strong degree of non-random mating identified in *W. bancrofti*, as well as the implications that this may have for the development and detection of anthelmintic resistance.

### Non-random infrapopulation mating

Our results suggest that adult *W. bancrofti* worms do not mate randomly within the infrapopulation. This is in agreement with ultrasonography studies that show adult parasites congregating in ‘worm nests’ along lymphatic vessels, which remain stable over time [44]. Spatial heterogeneity within the host may produce multiple reproducing populations within each infrapopulation, which would increase host microfilarial homozygosity. Evidence of an apparent relationship between β-tubulin genotype, the same gene analyzed by Schwab *et al.* [13], and female worm fertility in the related filaria *O. volvulus* has been reported by Bourguinat *et al.* [10]. If such a relationship exists in *W. bancrofti*, the excess within-host homozygosity reported above may result from the increased fertility of homozygous adult worms. Anthelmintic treatment, prior to the introduction of MDA for lymphatic filariasis, may also have increased non-random mating depending on the selective advantage that allele *Y* may confer to the parasite at the time of treatment.

### Parasite genetic differentiation between hosts

The degree of genetic differentiation in the parasite infrapopulation can shed insight into the microepidemiology of parasite transmission [19]–[23]. The metapopulation transmission dynamics of *W. bancrofti* will depend on the transmission efficiency and biting behaviour of the mosquito vector. *Anopheles gambiae sensu stricto* and *An. funestus* are thought to be the main vectors of *W. bancrofti* in Burkina Faso [45]. Hosts can acquire multiple L3 larvae during the same bite. Although density-dependent processes are known to operate on the uptake and development of *W. bancrofti* in *An. gambiae*, infective vectors will regularly transmit multiple related L3 larvae simultaneously [46]. Other mosquito vectors of *W. bancrofti* have even greater vector competence. For example, up to 32 L3 larvae were recovered from an experimental host after it was bitten by a single *Culex quinquefasciatus* [47], a main vector in East Africa. Mark-recapture studies and bloodmeal analysis indicate that various mosquito species appear to have high site fidelity, regularly biting multiple members of the same household [48],[49]. These aspects of *W. bancrofti* transmission increase the likelihood that a host will be infected with closely related parasites and will contribute to the observed genetic differentiation.

More generally, drug treatment may increase infrapopulation genetic heterogeneity, as those parasites within treated hosts which survive treatment may have a higher resistance allele frequency than those harboured within untreated hosts. In Burkina Faso, lymphatic filariasis is treated with albendazole and ivermectin. Evidence indicates that the albendazole plus ivermectin combination has some macrofilaricidal and reproductive effects (mainly associated with albendazole [41]), as well as the microfilaricidal effect (mainly associated with ivermectin). It is possible that a degree of the genetic differentiation between hosts observed in the untreated villages may have resulted from individual members of the community seeking, for instance, treatment for geohelminth infection prior to the introduction of GPELF.

### The spread of anthelmintic resistance

Population subdivision and non-random mating will influence the outcomes of selection under chemotherapeutic pressure in different ways, depending on the initial frequency of the allele under selection and the ecology of the infection. Before the rate of spread of drug resistant parasites can be predicted reliably and accurately, greater knowledge would be required regarding the number, linkage, dominance, and possible negative pleiotropic effects of putative resistance allele(s), as well as regarding the pharmacodynamic properties of the drugs administered singly and in combination. However, useful biological insights can be obtained from mathematical models that make reasonable assumptions concerning the above [50],[51].

If the resistance allele is recessive and it has a low initial frequency, inbreeding will increase parasite homozygosity and as a result, the spread of drug resistant worms across the parasite population (see Figure 6 and [50]). If drug resistance is a semi-dominant trait then parasite inbreeding will either increase or decrease the spread of drug resistance, depending on the efficacy of the drug against heterozygote parasites. Parasite genetic differentiation between hosts will also increase the spread of resistance even when the resistance allele is initially present at a very low frequency, as it increases the probability that male and female resistant worms will inhabit the same infrapopulation. This work is consistent with mathematical models of veterinary helminths which indicate that spatial heterogeneity and aggregated infections between hosts increase the spread of rare recessive genes [27],[28].

### The detection of anthelmintic resistance

The operation of a strong degree of parasite genetic differentiation between hosts reduces the prevalence of infection with drug resistant parasites and would therefore increase the number of hosts and parasites that should be sampled to detect and quantify the frequency of resistance-conferring alleles reliably. Even at high resistance allele frequencies, some hosts will have no phenotypic signs of resistance, particularly if the resistance allele is recessive, and therefore hosts respond to treatment. In practice the number of parasites that can be genotyped will be restricted, so surveys should carefully consider the sampling scheme they employ in order to maximise the accuracy of allele frequency estimates. Repeatedly sampling from the same host increases the chance of detecting a resistance mutation if it is present in that infrapopulation. However, sampling transmission stages from as many hosts as possible should be considered the optimum strategy, even in a population with low parasite genetic differentiation between hosts, as it reduces the chance of repeatedly sampling offspring of the same adult worm. Prior to the introduction of chemotherapy, studies investigating the presence and frequency of putative resistance markers through genotyping transmission stages alone should weight the number of samples they take per host by the host's infection intensity. However, after the start of chemotherapy the best sampling scheme will depend on the pharmacodynamics of the drug and the nature of the questions under investigation.

### Parasite elimination

For human helminth infections, the importance of parasite genetic differentiation between hosts stretches beyond population genetics and will influence the outcomes of parasite elimination campaigns such as the GPELF. The ability of a parasite species to persist in a host population following prolonged MDA will depend in part on the metapopulation dynamics of helminth transmission, the patterns of host compliance with treatment regimes and the pharmacodynamic properties of the drugs used. The aggregated nature of the passage of transmission stages between hosts will make parasite elimination harder to achieve by lowering the breakpoint density (the unstable equilibrium below which the parasite population will tend naturally to local extinction [52]), as overdispersion of parasites will result in fewer hosts with a single-sexed infection.
